# Polymorphisms in Iron Homeostasis Genes and Urinary Cadmium Concentrations among Nonsmoking Women in Argentina and Bangladesh

**DOI:** 10.1289/ehp.1205672

**Published:** 2013-02-15

**Authors:** Gerda Rentschler, Maria Kippler, Anna Axmon, Rubhana Raqib, Eva-Charlotte Ekström, Staffan Skerfving, Marie Vahter, Karin Broberg

**Affiliations:** 1 Department of Laboratory Medicine, Division of Occupational and Environmental Medicine, Lund University, Lund, Sweden; 2 Institute of Environmental Medicine, Karolinska Institutet, Stockholm, Sweden; 3 International Centre for Diarrhoeal Disease Research, Bangladesh (icddr,b), Dhaka, Bangladesh; 4 Department Women’s and Children’s Health, Uppsala University, Uppsala, Sweden

**Keywords:** ferritin, *SLC11A2*, *SLC40A1*, *TF*, *TFR2*, transferrin

## Abstract

Background: Cadmium (Cd) is a human toxicant and carcinogen. Genetic variation might affect long-term accumulation. Cd is absorbed via iron transporters.

Objectives: We evaluated the impact of iron homeostasis genes [divalent metal transporter 1 (*SLC11A2*), transferrin (*TF*), transferrin receptors (*TFR2* and *TFRC*), and ferroportin (*SLC40A1*)] on Cd accumulation.

Methods: Subjects were nonsmoking women living in the Argentinean Andes [*n* = 172; median urinary Cd (U-Cd) = 0.24 µg/L] and Bangladesh (*n* = 359; U-Cd = 0.54 µg/L) with Cd exposure mainly from food. Concentrations of U-Cd and Cd in whole blood or in erythrocytes (Ery-Cd) were measured by inductively coupled plasma mass spectrometry. Fifty polymorphisms were genotyped by Sequenom. Gene expression was measured in whole blood (*n* = 72) with Illumina DirectHyb HumanHT-12 v4.0.

Results: *TFRC* rs3804141 was consistently associated with U-Cd. In the Andean women, mean U-Cd concentrations were 22% (95% CI: –2, 51%), and they were 56% (95% CI: 10, 120%) higher in women with GA and AA genotypes, respectively, relative to women with the GG genotype. In the Bangladeshi women, mean U-Cd concentrations were 22% (95% CI: 1, 48%), and they were 58% (95% CI: –3, 157%) higher in women with GA and AA versus GG genotype, respectively [adjusted for age and plasma ferritin in both groups; *p*_trend_ = 0.006 (Andes) and 0.009 (Bangladesh)]. *TFRC* expression in blood was negatively correlated with plasma ferritin (*r*_S_ = –0.33, *p* = 0.006), and positively correlated with Ery-Cd (significant at ferritin concentrations of < 30 µg/L only, *r*_S_ = 0.40, *p* = 0.046). Rs3804141 did not modify these associations or predict *TFRC* expression. Cd was not consistently associated with any of the other polymorphisms evaluated.

Conclusions: One *TFRC* polymorphism was associated with urine Cd concentration, a marker of Cd accumulation in the kidney, in two very different populations. The consistency of the findings supports the possibility of a causal association.

Cadmium (Cd) is ubiquitous in the environment. Human exposure occurs via plant-derived foods and certain seafood, as well as from tobacco smoke (Olsson et al. 2002). Evidence of adverse health effects on kidney and bone has been reported in association with low-level environmental Cd exposure in adults (Åkesson et al. 2005; Engström et al. 2012), and recent studies have reported associations with hormone-related cancers (Åkesson et al. 2008; Julin et al. 2012).

In general, intestinal absorption of Cd is low: about 5% in adults (European Food Safety Authority 2009). However, because the half-time of Cd is very long (10–30 years), even small modifications in absorption rate can affect Cd accumulation and, in turn, its toxicity. Women are more prone than men to have low iron status, which is associated with a higher absorption of Cd in the intestines (Barany et al. 2005; Berglund et al. 1994; Gallagher et al. 2011; Kippler et al. 2007). As a consequence, women usually have higher Cd levels in blood (B-Cd) and urine (U-Cd) than men (Vahter et al. 2007). Twin studies have suggested genetic influences on Cd kinetics (Björkman et al. 2000; Whitfield et al. 2010), particularly in women (Björkman et al. 2000), but specific genetic mechanisms are uncertain. The rs28366003 G allele polymorphism of the *MT2A* metallothionein gene was associated with increased Cd in blood and reduced zinc (Zn) in serum in a Turkish study population (Kayaalti et al. 2011), and with higher Cd levels in kidney tissue collected at autopsy (Kayaalti et al. 2010), although the findings of the latter study need to be interpreted with caution because only one subject was homozygous for the G allele.

Uptake and transport of Cd is partly accomplished by proteins in the iron homeostasis system (Vesey 2010). The ferrous form of iron is taken up by the divalent metal transporter 1 [solute carrier family 11 (proton-coupled divalent metal ion transporters), member 2; *SLC11A2*, formerly *DMT1*], located in the apical membrane of duodenal enterocytes in the intestine. *SLC11A2* and its animal homologues have been shown to interact with Cd (Bressler et al. 2004; Tallkvist et al. 2001; Vesey 2010). In the cell, iron is reduced to the ferric form and exported to the blood by ferroportin 1 [solute carrier family 40 (iron-regulated transporter), member 1; *SLC40A1,* formerly *FPN1*] at the basolateral membrane. Troadec et al. (2010) reported a role for *FPN1* in transition metal efflux (Cd and Zn) in mouse bone marrow macrophages, and Park and Chung (2009) found that Cd exposure increased *FPN1* expression in macrophages. In blood, iron is bound to mobile transferrin (*TF*) and ferritin. An interaction between Cd and transferrin has been shown in buffered solution (Harris and Madsen 1988) and in rats (Huebers et al. 1987). Transferrin receptors (*TFRC* and *TFR2* genes) are highly homologous type II transmembrane proteins that help regulate intracellular iron by delivering iron from transferrin into the cytoplasm (Wang and Pantopoulos 2011). Increasing evidence indicates that single nucleotide polymorphisms (SNPs) in iron homeostasis genes have a functional impact on both ferritin and transferrin levels (Benyamin et al. 2009b; Constantine et al. 2009; Pichler et al. 2011) and on blood hemoglobin and red blood cell production (Benyamin et al. 2009a; Ganesh et al. 2009).

The main aim of this study was to elucidate whether SNPs in genes belonging to the iron homeostasis system are associated with biomarkers of Cd accumulation in humans. A secondary aim was to evaluate modification of gene expression as a potential mode of action.

## Methods

*Study areas and populations*. The ethical review committees of icddr,b, Bangladesh, the Health Ministry of Salta, Argentina, and Karolinska Institutet, Sweden, approved this study. Both oral and written informed consents were provided by all participants prior to the study.

Argentinean Andes. The Andean study participants were 172 women from San Antonio de los Cobres, a village in the northern Argentinean Andes (altitude 3,800 m; Table 1), who were part of a cross-sectional study on the health effects of toxic elements in drinking water and food. The sampling was performed in 2008 (Engström et al. 2011): Biological samples were collected during the daytime as nonfasting spot samples at the local health clinics and at the hospital in San Antonio de los Cobres. Peripheral blood and spot urine samples were collected and analyzed for Cd, and peripheral blood samples were collected for DNA and RNA extraction as well as isolation of plasma. None of the women were first-degree relatives. The main source of Cd exposure was probably food because only three of the women smoked, the drinking water Cd levels were low (< 0.17 µg/L Cd), and there was no industrial Cd pollution.

**Table 1:** Descriptive data and measured Cd and ferritin for the whole study population and the subsample for which gene expression was measured.

Variable	Bangladesh			Argentinean Andes					
	All women			All women			Gene-expressiona		
	n	Median	Range	n	Median	Range	n	Median	Range
Age (years)	359	26	14–44	172	36	12–80	72	34	12–65
Weight (kg)	357	44	30–72	172	57	37–100	72	56	37–87
Height (cm)	359	150	136–170	172	152	137–165	72	152	142–165
BMI (kg/m2)	357	19.4	14–29	172	25.1	16.4–40	72	24.0	16.4–36
B-Cd (µg/L)	—	—	—	172	0.36	0.17–1.1	72	0.32	0.17–1.1
Ery-Cd (µg/kg)b	234	1.2	0.35–4.7	172	0.75	0.36–2.1	72	0.68	0.37–1.9
U-Cd (µg/L)c	359	0.54	0.05–4.5	172	0.24	0.01–1.5	72	0.22	0.01–1.5
Plasma ferritin (µg/L)	355	29	2.8–200	166	52	4–1,200	70	48	4–320
10th–90th percentile			12–65			10–220			
5th–95th percentile			8–88			7–310			
Abbreviations: —, not measured; BMI, body mass index. aSubgroup included in gene expression analyses. bEry-Cd was measured in the Bangladeshi women and was estimated based on B-Cd concentrations for the Andean women. cNormalized for specific gravity.									

Bangladesh. The Bangadeshi study participants were female residents of Matlab, a rural area 53 km southeast of Dhaka who were included in a longitudinal study of the health effects of early-life exposure to toxic elements that was nested in the Maternal and Infant Nutrition Interventions in Matlab (MINIMat) trial. The study population and sampling procedures have been described in detail (Kippler et al. 2007, 2009). None of the women were smokers; thus, their Cd exposure probably originated from food (rice) (Kippler et al. 2010). Cd was measured in samples collected during early pregnancy, including urine (gestational week 8, range, 1–19 weeks) and blood (gestational week 4, range, 9–22 weeks). For the present study, we randomly sampled 500 of the 2,119 women enrolled during 2002. We were able to extract DNA from blood samples of 403 of these women, and measured Cd in blood and urine samples from 235 and 359 women, respectively.

*Analysis of Cd*. B-Cd in the Andean group and erythrocyte Cd (Ery-Cd) in samples from the Bangladeshi group were determined using inductively coupled plasma mass spectrometry (ICPMS) (Agilent 7500ce; Agilent Technologies, Tokyo, Japan), following microwave-assisted acid digestion (Kippler et al. 2009). The urine samples were diluted with 1% nitric acid, after which U-Cd was measured by the same ICPMS instrument with the collision/reaction cell system in helium mode to minimize interferences, particularly from molybdenum (Concha et al. 2010; Kippler et al. 2007). All the samples contained concentrations well above the limit of detection [LOD (which was 3× SD of the blank): 0.011 µg/L for B-Cd (Andes), < 0.1 µg/kg for Ery-Cd (Bangladesh), and < 0.05 µg/L for U-Cd (both study populations)]. To ascertain accuracy, commercially available reference materials with certified or recommended Cd concentrations were analyzed.

To enable comparisons between the two population groups, the B-Cd values measured in Andean women were adjusted to correspond to Ery-Cd, assuming an erythrocyte density of 1.055 g/mL and that 95% of Cd in whole blood is contained in erythrocytes (Nordberg et al. 2007). To account for the volume fractions of erythrocytes and plasma, we used the measured hemoglobin concentrations divided by 340 g/L, which is the mean reference value for hemoglobin in erythrocytes (Leon-Velarde et al. 2000; Lundh and Öhlin 1991). The Spearman correlation coefficient (*r*_S_) between measured B-Cd and estimated Ery-Cd in the Andes study population was 0.97 (*p* = 3.0 × 10^–7^).

Urine concentrations were normalized to the mean specific gravity of each population [EUROMEXRD712 clinical refractometer; EROMEX, Arnhem, Holland (Nermell et al. 2008)]: 1.020 g/mL in Andes and 1.012 g/mL in Bangladesh.

*Analysis of plasma ferritin*. Plasma ferritin was analyzed with an immunoassay (Cobas e601; Roche Diagnostics, Mannheim Germany; LOD 0.5 µg/L, imprecision 5.1%) for the Andean samples and with a radioimmunoassay [Diagnostic Products, San Diego, CA, USA (Kippler et al. 2007)] for the Bangladeshi samples.

*Genotyping of single nucleotide polymorphisms (SNPs)*. DNA was isolated from peripheral blood using the QIAmp DNA Blood Mini kit (QIAGEN, Hilden, Germany). Few iron-transporter gene SNPs have been shown to have a functional impact on gene expression or protein activity, and most of the nonsynonymous SNPs that have been identified are rare. Therefore, we selected SNPs that are markers of variation in larger segments of each of the five iron homeostasis genes (tagSNPs) based on linkage calculated using Haploview version 4.1 (Barrett 2009) for Asian population groups from Beijing and Tokyo [Han Chinese in Beijing, China (CHB) and Japanese in Tokyo, Japan (JPT)] included in Hapmap (http://hapmap.ncbi.nlm.nih.gov/citinghapmap.html.en). We also selected SNPs based on functional impact according to the literature or potential impact according to position and type of SNP (specifically, nonsynonymous SNPs that might affect the protein structure/transporter activity or 5´ SNPs at putative promoter sites that could influence the gene expression). Selected SNPs had a minor allele frequency (MAF) ≥ 5%, with the exception of 1 synonymous and 11 nonsynonymous SNPs from four genes according to dbSNP (http://www.ncbi.nlm.nih.gov/snp). Altogether, 58 SNPs were selected for genotyping (Sequenom Inc., San Diego, CA, USA).

The samples were considered adequate for genotyping if genotypes were reported for > 60% of the final SNPs. Two SNPs were excluded because their genotypes were automatically defined by the call algorithm in ≤ 90% of the adequate samples. No variants were detected in 6 of the remaining SNPs, leaving 50 informative SNPs for analysis [see Supplemental Material, Table S1, and for excluded SNPs, Table S2 (http://dx.doi.org/10.1289/ehp.1205672)]. Deviations from the Hardy–Weinberg equilibrium (HWE) were tested using chi-square analysis. None of the SNPs showed Hardy–Weinberg disequilibrium in both populations. Two SNPs demonstrated disequilibrium in one of the populations and were included in further analysis (see Supplemental Material, Table S1).

Transcription factor sites that may be created or disrupted by SNPs were identified using the ElDorado database (version 08-2011; Genomatix software suite; http://www.genomatix.de/en/index.html) [for a list of transcription factor binding sites modified by SNPs, associated with Cd or ferritin, see Supplemental Material, Table S3 (http://dx.doi.org/10.1289/ehp.1205672)].

*RNA collection and gene expression analysis*. Peripheral blood was collected in PAX tubes (PreAnalytiX GmbH, Hombrechtikon, Switzerland). All samples were frozen and stored at –20°C after ≤ 24 hr at room temperature. RNA was extracted with the PAXgene Blood RNA kit (PreAnalytiX GmbH) and stored at –80°C. RNA concentration and purity were evaluated on a Nanodrop spectrophotometer (Wilmington, DE, USA). Adequate RNA integrity [RNA integrity number (RIN) > 7.5] was confirmed using a Bioanalyzer 2100 (Agilent, Santa Clara, CA, USA). The gene expression analysis included 72 randomly selected Andean women, none of whom were first-degree relatives. For the whole-genome gene expression analysis, DirectHyb HumanHT-12 version 4.0 (Illumina, San Diego, CA, USA) was used according to the manufacturer’s instructions [for a list of probes, see Supplemental Material, Table S4 (http://dx.doi.org/10.1289/ehp.1205672)]. Background signals were filtered from the gene expression by BioArray Software Environment (BASE) (Vallon-Christersson et al. 2009).

*Statistical analysis*. The Andean and Bangladeshi study groups were analyzed separately and then compared. Linkage disequilibrium (LD) analyses were performed using Haploview version 4.1 (Barrett 2009). When the frequency of a homozygote genotype was very low (< 9 individuals), this group was pooled with the heterozygotes. In these cases, we visually inspected scatter plots of all associations between SNPs and Cd/ferritin concentrations before pooling, to ensure that pooling was justified on the basis of the data.

**Figure 1:**
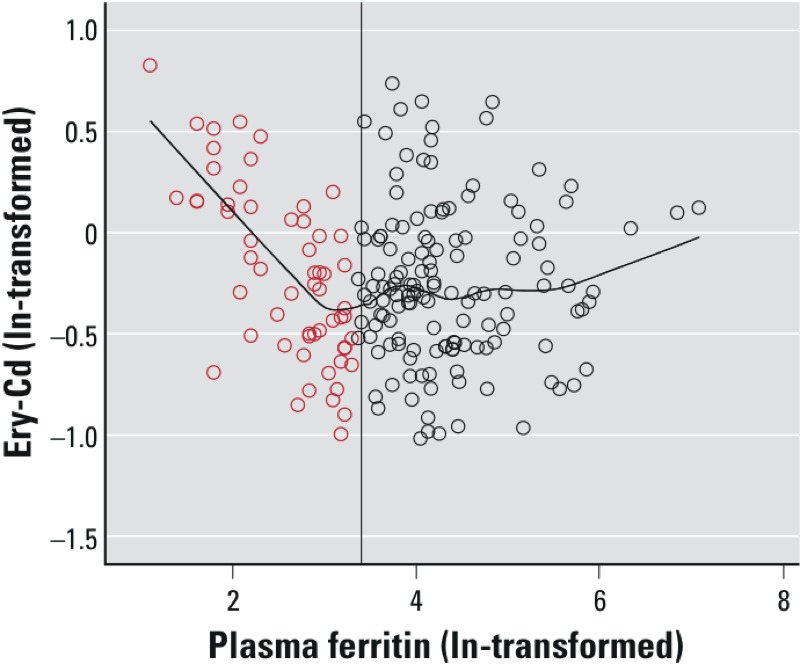
Scatter plot showing the association between estimated Cd in erythrocytes (ln-transformed, based on measured concentrations in whole blood) and plasma ferritin (ln-transformed) in Andean women. The reference line is drawn at 3.4, corresponding to a plasma ferritin concentration of 30 µg/L in normal scale. The fit line is calculated by the LOESS-method with biweight kernel in order to emphasize the relation between Ery-Cd and plasma ferritin. Red circles represent the subgroup considered to have low iron stores in this study.

Associations between participant characteristics and exposure markers were evaluated using Spearman correlation coefficients. Associations between genotypes (independent variables) and natural log (ln)-transformed U-Cd or Ery-Cd (dependent variables) were estimated using multivariable-adjusted regression with the general linear model procedure to allow the three possible genotypes for each SNP to be modeled without assuming additive effects. All models were adjusted for age. *p*-Values for trend were calculated entering genotype as a continuous variable into the model. Plasma ferritin was considered to be a potential effect modifier. Because ferritin may be influenced by menopause, we stratified by age (45 years as a proxy cut-off for pre- and postmenopausal age). We also stratified according to ferritin values (< 30 or ≥ 30 µg/L) using data-derived cut-offs based on Ery-Cd in relation to ferritin (Figure 1). A fit line was calculated by LOESS method with biweight kernel for the relation between Ery-Cd and plasma ferritin. Here, we present relative differences in Ery-Cd and U-Cd according to genotype, using the most common genotype in Bangladesh as the reference to facilitate comparisons between the two study populations.

We analyzed correlations between U-Cd or Ery-Cd and ferritin concentrations and gene expression data using Spearman correlation coefficients. Associations between SNPs and gene expression were analyzed using Kruskal–Wallis tests.

Calculations were made with PASW Statistics version 18 (http://www.spss.com.hk/statistics/). Nominal statistical significance was determined as *p* < 0.05 (two-tailed). Multiple comparison-adjusted *p*-values were calculated for each population and each outcome marker using the false discovery rate (FDR) procedure [R version 2.14.2 (http://www.r-project.org/)]. The numbers of independent comparisons were based on the numbers of SNPs that were not in LD (*r*^2^ < 80%), resulting in 24 tests for the Andean and 29 for the Bangladeshi populations.

## Results

*Characteristics of study participants*. Descriptive data of the women and concentrations of exposure markers and ferritin are listed in Table 1. The Andean women were older than the Bangladeshi (median 36 vs. 26 years; *p* < 0.001) and their median plasma ferritin concentration was almost twice as high (median 52 µg/L vs. 29 µg/L). Ery-Cd in the Andean women (median 0.75 µg/kg estimated, based on B-Cd as described previously) was lower than in the Bangladeshi women (1.2 µg/kg), as was U-Cd (0.24 µg/L vs. 0.54 µg/L). Andean women included in the gene expression analysis were similar to the Andean study population as a whole (Table 1).

Ery-Cd and U-Cd were positively correlated, and both increased with age (Table 2). Among all the Andean women, U-Cd, but not B-Cd or Ery-Cd, was weakly positively correlated with ferritin. However, for Andean women with ferritin concentrations of < 30 µg/L (*n* = 50), Ery-Cd was negatively correlated with ferritin (*r*_S_ = –0.58, *p* = 0.000011; Figure 1). For Bangladeshi women, Ery-Cd, but not U-Cd, was significantly negatively correlated with ferritin (*r*_S_ = –0.15, *p* = 0.02).

**Table 2:** Spearman’s correlation coefficients (rS) between age, Ery-Cd, B-Cd, U-Cd, and ferritin in plasma in the Andean and Bangladeshi populations.

Covariate	Ery-Cda		B-Cd		U-Cd		Ferritin	
	Andes	Bangladesh	Andes	Bangladesh	Andes	Bangladesh	Andes	Bangladesh
Age								
rS	0.32	0.18	0.40	—	0.42	0.23	0.50	–0.02
p	1.5 × 10–5	0.006	5.8 × 10–8		8.7 × 10–9	1.3 × 10–5	1.0 × 10–11	0.8
n	172	234	172	—	172	359	166	355
Ery-Cd								
rS			0.97		0.42	0.53	–0.09	–0.15
p			3.0 × 10–7		1.2 × 10–8	1.4 × 10–24	0.2	0.001
n			172		172	234	166	230
B-Cd								
rS					0.45	—	0.03	—
p					5.0 × 10–10		0.7	
n					172	—	166	—
U-Cd								
rS							0.21	–0.08
p							0.008	0.1
n							166	355
—, Not measured. aEry-Cd was measured in the Bangladeshi women and was estimated based on B-Cd concentrations for the Andean women.								

Body mass index (BMI) was significantly correlated with age in the Andean population (*r*_S_ = 0.42; data not shown). Therefore, we adjusted for age only in the analysis of gene × environment interactions.

Allelic frequencies differed by ≥ 30% between the Andean and Bangladeshi study populations for *SLC11A2* rs149411 (C allele 75% vs. 37%, respectively) and rs224575 (G allele 75% vs. 37%) and for *TF* rs2280673 (C allele vs. 15% vs. 48%). The *TFRC* rs3804141 A allele was carried by 26% of the Andean women and 14% of the Bangladeshi women. [See Supplemental Material, Table S1 (http://dx.doi.org/10.1289/ehp.1205672).] All of the 50 SNPs included in analyses were in HWE in the Bangladeshi population, but *TFRC* rs3804141 and *TFR2* rs7385804 were not in HWE in the Andean population.

*Iron-related genes and Cd*. *TFRC* rs3804141 was associated with U-Cd in the same direction in both study populations: Women with the GA or AA genotypes had significantly higher U-Cd than women with the GG genotype, with the strongest associations estimated for the AA genotype (Table 3). In the Andeans, mean U-Cd concentrations were 22% (95% CI: –2, 51%) and 56% (95% CI: 10, 120%) higher in women with GA and AA genotypes, respectively, relative to women with the GG genotype (adjusted for age and ferritin). In Bangladesh, mean U-Cd concentrations were 22% (95% CI: 1, 48%) and 58% (95% CI: –3, 157%) higher in women with GA and AA versus GG genotype, respectively. After FDR adjustment, trend *p*-values became nonsignificant (*p*_adjusted_ = 0.07 in the Andeans and *p*_adjusted_ = 0.26 in the Bangladeshi). For *TFRC* rs3804141 genotypes and Ery-Cd, there was no association with GA and a very weak positive association with AA in Andean women and only a weak positive association with GA and AA combined in Bangladeshi women.

**Table 3:** Relative changes of Ery-Cd and U-Cd concentrations depending on genotypes of TFRC rs3804141.

Gene, SNP	Population	Genotypea	Ery-Cd			U-Cd		
			n	Relative change (95% CI)	pTrend	n	Relative change (95% CI)	pTrend
TFRC
rs3804141	Andes	GG	94	1		98	1
		GA	52	1.01 (0.89, 1.14)		53	1.22 (0.98, 1.51)
		AA	15	1.11 (0.91, 1.35)	0.6	16	1.56 (1.10, 2.20)	0.006
	Bangladesh	GG	179	1		267	1
		GA	55	1.08 (0.92, 1.27)		82	1.22 (1.01, 1.48)
		AA	—		0.3	10	1.58 (0.97, 2.57)	0.009
—, Combined with the heterozygote genotype because the frequency of the homozygote genotype was very low (AA n = 5). Linear regression models adjusted for age and plasma ferritin. aReference genotype, GG, is the most common homozygote in Bangladesh.								

Among Andean women, the association between rs3804141 genotype and U-Cd was evident among women < 45 years of age [median concentration for GA + AA genotypes (*n* = 52) 41% higher (95% CI: 10, 80%) than the GG genotype (*n* = 52); *p* = 0.005), but not among the older women [U-Cd in GA + AA genotypes (*n* = 17) 1% higher (95% CI: –30, 60%) than in GG genotype (*n* = 36); *p* = 0.99]. Andean women with ferritin concentrations of < 30 µg/L had a similar, although nonsignificant association between U-Cd and genotype as the whole study population [GA + AA (*n* = 23) had 38% higher (95% CI: –7, 105%) U-Cd than those with GG (*p* = 0.1)]. Among Bangladeshi women with ferritin concentrations of < 30 µg/L, GA + AA (*n* = 43) had 38% higher (95% CI: 7, 76%) U-Cd than GG (*p* = 0.01).

*TFRC* rs3804141 was only in weak LD with the other eight *TFRC* SNPs: *r*^2^ < 26% among Andean women and *r*^2^ < 16% among Bangladeshi women. [For a list of the nine *TFRC* SNPs included in the analysis, see Supplemental Material, Table S1 (http://dx.doi.org/10.1289/ehp.1205672)].

U-Cd concentration was associated with SNPs in other genes, but only in the Andean women. For *TF* rs3811647 and the two SNPs that were in LD with it [rs12595 (*r*^2^ = 92%) and rs4459901 (*r*^2^ = 78%)], mean U-Cd concentrations were significantly lower in association with heterozygote versus reference genotypes (Table 4). However, although trend *p*-values were significant, differences in mean U-Cd concentrations were smaller for homozygous variant genotypes than heterozygotes, and associations were not significant after FDR adjustment. *TFR2* rs7385804 also was negatively associated with U-Cd in the Andeans, based on the estimated difference for combined CA and CC genotypes relative to the reference AA genotype (*p* = 0.0004; FDR adjusted *p* = 0.0096; Table 4). As previously noted, neither *TFR2* rs7385804 nor *TFRC* rs3804141 were in HWE in Andean women. None of the *TF* or *TFR2* SNPs were significantly associated with Ery-Cd (in either population) or with U-Cd in the Bangladeshi group.

**Table 4:** Study population-specific relative changes of Ery-Cd and U-Cd concentrations depending on TF and TFR2 SNPs.

Gene, SNP	Population	Genotypea	Ery-Cd			U-Cd		
			n	Relative change (95% CI)b	p-Value	n	Relative change (95% CI)	p-Value
TF								
rs3811647	Andes	GG	32	1	0.5	32	1	0.009
		AG	82	0.91 (0.78, 1.06)		82	0.65 (0.50, 0.86)
		AA	54	0.91 (0.78, 1.07)		54	0.76 (0.57, 1.00)
	Bangladesh	GG	76	1	0.5	109	1	0.2
		AG	116	1.01 (0.87, 1.18)		181	0.93 (0.77, 1.11)
		AA	34	1.14 (0.92, 1.41)		58	1.14 (0.89, 1.45)
rs12595	Andes	AA	30	1	0.5	30	1	0.01
		GA	89	0.91 (0.78, 1.07)		89	0.66 (0.50, 0.86)
		GG	50	0.94 (0.80, 1.11)		50	0.78 (0.58, 1.05)
	Bangladesh	AA	82	1	0.5	116	1	0.1
		GA	114	1.0 (0.87, 1.17)		182	0.91 (0.76, 1.09)
		GG	36	1.1(0.91, 1.38)		59	1.13 (0.89, 1.45)
rs4459901	Andes	TT	40	1	0.9	40	1	0.036
		TC	83	0.97 (0.84, 1.12)		83	0.72 (0.56, 0.92)
		CC	46	1.01 (0.86, 1.18)		46	0.79 (0.60, 1.04)
	Bangladesh	TT	91	1	0.7	143	1	0.5
		TC	111	1.04 (0.87, 1.34)		158	1.05 (0.88, 1.25)
		CC	31	1.08 (0.90, 1.21)		57	1.15 (0.90, 1.47)
TFR2								
rs7385804	Andes	AA	114	1	0.1	114	1	0.0004
		CA/CCc	52	0.91 (0.81, 1.02)		52	0.68 (0.55, 0.84)
	Bangladesh	AA	102	1	0.6	152	1	0.4
		CA	92	0.96 (0.83, 1.12)		150	0.89 (0.74, 1.06)
		CC	40	0.91 (0.75, 1.11)		57	0.98 (0.77, 1.25)
Linear regression models adjusted for age and plasma ferritin. aReference genotype is the most common homozygote in Bangladesh. bEry-Cd was measured in the Bangladeshi women and was estimated based on B-Cd concentrations for the Andean women. cThe CA and CC genotypes were combined because the frequency of the homozygote genotype was very low (CC n = 9).								

All of the SNPs that were associated with Cd biomarkers (rs3804141, rs3811647, rs12595, rs4459901, and rs7385804) or ferritin (rs8177186) potentially affect transcription factor binding sites [see Supplemental Material, Table S3 (http://dx.doi.org/10.1289/ehp.1205672)]. For example, the A allele of *TFRC* rs3804141 creates a potential binding site for a transcription factor from the homeobox/homeodomain family, whereas the site is abolished for carriers of the G allele.

*Iron-related genes and ferritin*. There was no significant association between *TFRC* rs3804141 and ferritin (data not shown) in either study population. However, among Andean women < 45 years of age, the A-allele carriers had 36% lower ferritin concentration (95% CI: –60, –10) than women with GG (*p* = 0.04). In the Bangladeshi group, there was no association between genotype and ferritin.

Individuals with *TF* rs8177186 GT (*n* = 99) or TT (*n* = 14) genotypes showed significantly higher ferritin concentrations than those with GG in the Bangladeshi women (7%; 95% CI: –10, 30% and 75%; 95% CI: 20, 160%, respectively), but not in the Andeans (data not shown). This SNP affects several putative transcription factor binding sites [see Supplemental Material, Table S3 (http://dx.doi.org/10.1289/ehp.1205672)].

*Expression of iron-related genes, Cd, and ferritin among Andean women*. *TFRC, SLC11A2,* and *SLC40A1* showed the highest gene expression levels, and the largest range, and they were significantly positively correlated [see Supplemental Material, Table S4 (http://dx.doi.org/10.1289/ehp.1205672)]. *TFRC* expression was significantly negatively correlated with ferritin (*r*_S_ = –0.33; Table 5). There were no other significant correlations between gene expression and ferritin or Cd in the Andean population as a whole. However, among 26 Andean women with ferritin concentrations of < 30 µg/L, *TFRC* expression was significantly positively correlated with Ery-Cd (*r*_S_ = 0.40; *p* = 0.046) and negatively correlated with plasma ferritin (*r*_S_ = –0.43; *p* = 0.03), *SLC11A2* expression was negatively correlated with U-Cd (*r*_S_ = –0.42; *p* = 0.03), and *SLC40A1* expression was negatively correlated with plasma ferritin (*r*_S_ = –0.49; *p* = 0.01).

**Table 5:** Spearman correlations (rS) of gene expression versus Cd exposure markers in Andean women [all (*n* = 70–72) and in those with low ferritin (< 30 µg/L) only (*n* = 26)].

Gene		Ery-Cd		B-Cd		U-Cd		Plasma ferritin	
		All	Low ferritin	All	Low ferritin	All	Low ferritin	All	Low ferritin
TF	rS	0.02	0.20	–0.06	0.04	0.05	0.03	–0.04	–0.42*
TFR2	rS	0.15	0.23	0.16	0.21	0.08	0.36	–0.036	–0.36
TFRC	rS	0.09	0.40*	0.04	0.30*	–0.04	0.22	–0.33**	–0.43*
SLC11A2	rS	0.13	0.16	0.16	0.16	–0.01	–0.42*	0.08	0.07
SLC40A1a	rS	0.05	0.24	–0.0.01	0.08	–0.04	0.06	–0.18	–0.49*
aExpression probe ILMN_2053103. *p < 0.05. **p < 0.01.									

*TFRC* rs3804141 was not associated with *TFRC* gene expression, and it did not appear to modify the relation between *TFRC* expression and ferritin (data not shown). However, carriers of the *TF* rs8177186 T-allele had significantly higher *TF* expression than women with the more common GG genotype (median 118 vs. 111; *p* = 0.050).

## Discussion

The rs3804141 *TFRC* gene SNP was significantly associated with U-Cd concentrations in both study populations. Higher U-Cd concentrations with increasing numbers of A alleles suggests that the variant allele may be a cause of increased Cd accumulation in the kidneys. Several other populations have A allele frequencies of ~ 20% (see http://www.ncbi.nlm.nih.gov/projects/SNP), as were also found in the present study populations (26% and 14% in Andean and Bangladeshi women, respectively). The *TFRC* gene has not been linked to Cd concentrations previously.

Among Andean women, rs3804141 was associated with U-Cd only among women < 45 years of age (who were assumed to be premenopausal); rs3804141 was also associated with ferritin in the Andean women. These findings suggest that regulation of iron uptake may play a role in the association between rs3804141 and Cd. The lack of association between rs3804141 and ferritin in the Bangladeshi women might be explained by undernourishment (Kippler et al. 2009) and pregnancy, because both conditions may lead to up-regulation of iron and Cd absorption (Åkesson et al. 1998, 2002).

Associations between Cd and SNPs in the other major genes regulating iron absorption (i.e., *TFR2* and *TF*) should be interpreted with caution because the associations were not consistent between the populations.

The relatively small number of study subjects, especially from the Andes, was a limitation; it resulted in an insufficient number of homozygote variant carriers for many SNPs. We had only one significant association after adjusting for multiple comparisons, which probably relates to the small study size as well. A main strength of this study was the wide range of Cd exposure; although none of the study sites had known sources of Cd pollution. Furthermore, the study populations were homogeneous for several potential confounders that may influence Cd concentrations (e.g., being nonsmokers, living in areas without industrial Cd pollution). We made several statistical sensitivity analyses. We stratified for age (45 years as a proxy for the pre/postmenopausal cut-off) in the Andean group because the population included women from 12 to 80 years of age and because menopause might influence ferritin and other factors that might confound associations between genotypes and Cd metabolism. The women in the Andes had markedly higher iron status (measured as plasma ferritin) than those in Bangladesh. The likely reasons are the high altitude and their meat-based diet, and possibly also that the Bangladeshi women were younger (14–44 years of age) and pregnant. Despite these differences, Ery-Cd was negatively correlated with ferritin in both populations, especially when ferritin concentrations were < 30 µg/L, similar to findings in previous studies (Berglund et al. 1994). The lower iron status in Bangladesh, together with higher intake of Cd via the rice-based diet, may explain the higher mean Cd concentrations in Bangladeshi women compared with Andean women (Kippler et al. 2007, 2009). We measured gene expression in whole blood, where *TF*, *TFRC*, *TRF2*, *SLC40A1,* and *SLC11A2* genes are not highly expressed (Uhlen et al. 2010; Wu et al. 2009); therefore, the gene expression analyses should be repeated based on expression in tissues where these genes are more highly expressed.

Consistent results for both study populations were only found for one intronic *TFRC* SNP: The A allele of rs3804141 was associated with increased U-Cd concentrations. Although the associations became nonsignificant after FDR-adjustment, the magnitudes of the associations were similar between the two very different study populations. Cd accumulates in the kidney with a long half-life; thus hypothetically, differences would increase with rising age. However, in this study, the strongest associations were actually found before menopause, which might be related to the iron status usually increasing after menopause, and thus, Cd accumulation associated with low iron status progresses more slowly. Still, other mechanisms might be involved to explain the differences related to age. U-Cd is the biomarker used to measure long-term Cd exposure. Genetic effects on Cd accumulation in erythrocytes are probably more difficult to identify because erythrocytes only reflect exposure within the last 3 months because of their limited life span.

We have to acknowledge the possibility that the associations between rs3804141 and U-Cd may have reflected the effects of an unmeasured variant in LD with this SNP. In addition, the findings may have been spurious in the Andean women because rs3804141 was not in HWE in this group. One way to show whether the association of rs3804141 and Cd accumulation is spurious or causal, would be to expose erythrocyte precursor cells, from donors with different genotypes, to Cd and to measure for differences in cellular Cd concentrations between the genotypes.

There were some study population-specific associations. In the Andean population group, variant genotypes of several *TF* SNPs in LD with rs3811647 were associated with lower U-Cd concentrations. In contrast to the findings of Constantine et al. (2009) and Pichler et al. (2011), none of those were associated with ferritin concentrations in our study. The C-allele of *TFR2* rs7385804 was associated with lower U-Cd concentration, also after adjusting for multiple comparisons, and it has been associated with lower serum iron (Pichler et al. 2011) and lower hematocrit (Ganesh et al. 2009) but not with ferritin or transferrin concentrations. The association of rs7385804 with markers of iron metabolism suggests a true effect of rs7385804 on U-Cd. However, this should be confirmed because the SNP was not in HWE in the Andean group.

## Conclusions

One SNP in *TFRC* was associated with U-Cd concentration, a marker of Cd accumulation, in two very different study populations of women. However, further studies are needed to confirm the association. Moreover, studies should be performed on men as well as women.

## Supplemental Material

(496 KB) PDF
