# Supplementary material for: Polymorphisms in Iron Homeostasis Genes and Urinary Cadmium Concentrations among Nonsmoking Women in Argentina and Bangladesh
Source: Environ Health Perspect. 2013 Feb 15;121(4):467–72. doi: 10.1289/ehp.1205672 (PMC3620753; doi:10.1289/ehp.1205672)
Supplement: (496 KB) PDF [file ehp.1205672.s001.pdf]

Supplemental Material

Polymorphisms in Iron Homeostasis Genes and Urinary Cadmium Concentrations among Nonsmoking Women in Argentina and Bangladesh

Gerda Rentschler, Maria Kippler, Anna Axmon, Rubhana Raqib, Eva-Charlotte Ekström, Staffan Skerfving, Marie Vahter, Karin Broberg

Table of Contents: **Page**

Supplemental Material, Table S1 **2**
Genes and polymorphisms that were successfully genotyped.

Supplemental Material, Table S2
Polymorphisms that were genotyped but later excluded from the data evaluation. **4**

Supplemental Material, Table S3 **5**
SNP-related modification of transcription factor binding sites

Supplemental Material, Table S4 **7**
Gene expression data for iron-related genes in the Andean women

Supplemental Material, Table S1. Genes and polymorphisms that were successfully genotyped (quality requirements: at least 90% of samples with clearly defined genotypes[a)]).

Gene<br>Unigene Nr[b]<br>Protein name<br>Location	rs nr[c]	Polymorphism type[d]	Allele frequencies Argentinean Andes	QC[a] (%)	Allele frequencies Bangladesh	QC[a] (%)
SLC11A2	rs149411	Intron C>T	75/25	96	37/63	99
Hs.505545	rs224572	Intron G>A	41/59	96	28/72	94
DMT1	rs224574	Intron C>T	1/99	98	10/90	99
NRAMP2	rs224575	Intron G>A	75/25	97	37/63	99
Chr 12q13	rs364627	Intron G>A	1/99	96	10/90	93
	rs407135	Intron C>A	8/92	97	34/66	100
	rs1005559	Intron T>A	7/93	97	24/76	96
	rs2269683	Intron C>T	34/66	96	9/91	99
	rs3809320	Upstream T>A	35/65	97	9/91	96
	rs6580779	5’ UTR G>T	1/99	96	10/90	95
	rs12366756	Intron G>A	7/93	98	24/76	99
	rs12830073	Intron T>C	6/94	96	24/76	90
SLC40A1	rs1123110	Intron C>T	12/88	95	34/66	97
Hs.643005	rs1439816	Intron C>G	12/88	97	22/78	100
FPN1	rs4145237	Intron C>G	15/85	97	33/67	99
Chr 2q32	rs4667287	Intron C>A	14/86	98	19/81	100
	rs11884632	Intron C>G	0/100	98	13/87	98
TF	rs12595	Intron G>A	58/43	98	41/59	99
Hs.518267	rs1049296	S589P T>C	4/96	98	21/79	99
Transferrin	rs1130459	5’ UTR A>G	6/94	98	25/75	97
Chr 3q22.1	rs1799852	L247L T>C	12/88	97	21/79	99
	rs2280673	Intron C>A	15/85	98	48/52	98
	rs2715627	Intron C>T	3/97	97	15/85	99
	rs3811647	Intron A>G	58/42	97	42/58	96
	rs4241357	Intron G>T	29/71	97	28/72	100
	rs4355280	Intron G>A	58/42	96	53/47	96
	rs4428180	Intron G>A	37/63	96	23/77	97
	rs4459901	Intron C>T	54/46	98	37/63	99
	rs4532136	Intron A>G	12/88	98	18/82	99
	rs6785596	Intron A>T	3/97	98	15/85	99
	rs6796795	Intron G>A	3/97	97	15/85	100
	rs8177184	Intron G>A	36/64	98	22/78	99
	rs8177186	5’ UTR T>G	12/88	96	18/82	97
	rs8177190	Intron T>C	25/75	97	9/91	97
	rs8177191	Intron A>G	2/98	98	7/93	99
	rs8177213	Intron C>A	27/73	97	14/86	96
	rs8177235	Intron A>G	14/86	97	16/84	97
	rs41298977	A76V T>C	0/100	98	0/100	99

Supplemental Material, Table S1 (cont.)

Gene<br>Unigene Nr[b]<br>Protein name<br>Location	rs nr[c]	Polymorphism type[d]	Allele frequencies Argentinean Andes	QC[a] (%)	Allele frequencies Bangladesh	QC[a] (%)
TFR2	rs7385804	Intron C>A	18/82	96 (*)	37/63	100
Hs.544932	rs10247962	Intron G>A	2/98	98	24/76	99
TFR2	rs34242818	I238M G>C	0/100	98	1/99	98
Chr 7q22						
TFRC	rs2284889	Intron T>C	27/73	97	50/50	98
Hs.529618	rs3736651	Intron T>A	43/57	96	21/79	99
TFRC	rs3761717	Intron C>G	73/27	97	49/51	94
Chr 3q29	rs3804141	Intron A>G	26/74	97 (*)	14/86	100
	rs9859260	Intron G>A	73/27	97	50/50	95
	rs9859401	Intron T>G	27/73	97	49/51	95
	rs11185506	Intron C>G	27/73	97	49/51	98
	rs13072608	Intron G>A	73/27	97	49/51	96
	rs41301381	L212V G>C	0/100	98	0/100	100

[a] QC percent represents the percentage of samples with clearly defined genotypes (based on Sequenom mass spectrometry analysis). None of the SNPs showed Hardy-Weinberg disequilibrium in both populations. SNPs that demonstrated disequilibrium in one of the populations are marked with (*).
[b] Unigene accession number from NCBI (National Center for Biotechnology Information) Unigene Database (website: http://www.ncbi.nlm.nih.gov/entrez/query.fcgi?db=unigene).
[c] rs numbers from NCBI SNP Database (website: http://www.ncbi.nlm.nih.gov/SNP).
[d] When applicable, amino acid position/gene region is denoted. The rare allele in Bangladesh is denoted first.

Supplemental Material, Table S2. Polymorphisms that were genotyped but later excluded from the data evaluation.

Gene	rs nr[a]	Polymorphism type[b]	QC[c] Argentinean Andes (%)	QC[c] Bangladesh (%)	Reason for exclusion
SLC11A2	rs224446	3' UTR A>G	82	88	too low analytical quality
	rs17216051	T298I C>T	98	99	no variants detected
	rs17216086	W500R T>C	97	99	no variants detected
	rs17222449	Q290R A>G	97	99	no variants detected
	rs17222470	H62Y C>T	98	99	no variants detected
SLC40A1	rs11568350	H248Q T>G	97	99	no variants detected
	rs45606432	L443P T>C	97	100	no variants detected
TFRC	rs3817672	S142G A>G	78	89	too low analytical quality

[a] rs numbers from NCBI SNP Database (website: http://www.ncbi.nlm.nih.gov/SNP).
[b] When applicable, amino acid position/gene region is denoted.
[c] QC percent represents the percentage of samples with clearly defined genotypes (based on Sequenom mass spectrometry analysis).

Supplemental Material, Table S3. SNP-related modification of transcription factor binding sites according to the ElDorado database[a]. SNPs analyzed were associated with concentrations of Cd biomarkers or ferritin (see Result section of the main text).

Gene<br>SNP	Allele	Effect	Site affected<br>Family/Factor	Description
TFRC				
rs3804141	G -> A	new	HOXC/HOX_PBX	HOX/PBX binding sites
	G -> A	new	SNAP/PSE02	Proximal sequence element (PSE) of RNA polymerase III-transcribed genes
TF				
rs12595	A -> G	lost	FKHD/HNF3.01	Hepatocyte nuclear factor 3 (alpha, beta) (FOXA1, FOXA2)
	A -> G	new	HOMF/HMX2.02	Hmx2/Nkx5-2 homeodomain transcription factor
	A -> G	new	MYT1/MYT1.01	MyT1 zinc finger transcription factor involved in primary neurogenesis
rs3811647	G -> A	new	NR2F/TR2.01	Nuclear hormone receptor TR2, DR5 binding sites
rs4459901	T -> C	new	CREB/CREB.02	cAMP-responsive element binding protein
	T -> C	new	CREB/ATF1.01	Activating transcription factor 1
	T -> C	new	CREB/CREB1.01	cAMP-responsive element binding protein 1
	T -> C	new	E4FF/E4F.01	GLI-Krueppel-related transcription factor, regulator of adenovirus E4 promoter
	T -> C	new	HIFF/HIF1.01	Hypoxia induced factor-1 (HIF-1)
	T -> C	lost	LHXF/ISL1.01	Pancreatic and intestinal lim-homeodomain factor
	T -> C	lost	PDX1/PDX1.01	Pdx1 (IDX1/IPF1) pancreatic and intestinal homeodomain TF
	T -> C	lost	RXRF/RAR_RXR.03	Retinoic acid receptor / retinoid X receptor heterodimer, DR5 sites

Supplemental Material, Table S3 (cont.)

Gene<br>SNP	Allele	Effect	Site affected<br>Family/Factor	Description
rs8177186	G -> T	new	CART/PHOX2.01	Phox2a (ARIX) and Phox2b
	G -> T	new	CART/CART1.01	Cart-1 (cartilage homeoprotein 1)
	G -> T	new	PARF/DBP.01	Albumin D-box binding protein
	G -> T	lost	TEAF/TEAD.01	TEA domain-containing factors, transcriptional enhancer factors 1,3,4,5
TFR2				
rs7385804	C -> A	new	BCDF/PCE1.01	Photoreceptor conserved element 1
	C -> A	new	DLXF/DLX3.01	Distal-less 3 homeodomain transcription factor
	C -> A	new	HBOX/GSH2.01	Homeodomain transcription factor Gsh-2
	C -> A	new	HOMF/MSX2.01	Muscle segment homeobox 2, homologue of Drosophila (HOX 8)
	C -> A	new	NKXH/NKX25.02	Homeodomain factor Nkx-2.5/Csx, tinman homolog low affinity sites
	C -> A	new	OCT1/OCT1.06	Octamer-binding factor 1
	C -> A	lost	SORY/HBP1.01	HMG box-containing protein 1
	C -> A	lost	TEAF/TEAD.01	TEA domain-containing factors, transcriptional enhancer factors 1,3,4,5

[a] Version 08-2011 of the Genomatix software suite (http://www.genomatix.de/en/index.html)

Supplemental Material, Table S4. Gene expression data for iron-related genes measured in peripheral blood in the Andean women (N=72).

Gene	Probe	Probe location	Expression[a] Median (Range)	Spearman correlation coefficients (p-values)				
				TF[b]	*TFR2*[b]	*TFRC*[b]	*SLC11A2*[b]	*SLC40A1*[b]
TF	ILMN_1768425	exon 17	112 (86-147)					
TFR2	ILMN_1724738	exon 18	111 (91-138)	0.18 (0.1)				
TFRC	ILMN_1674243	exon 19	294 (168-773)	0.05 (0.7)	-0.12 (0.3)			
SLC11A2	ILMN_1745034	exon 19b	132 (101-201)	-0.19 (0.1)	-0.21 (0.08)	0.32 (0.006)		
SLC40A1	ILMN_1761833	exon 8	255 (117-570)	0.15 (0.2)	0.09 (0.4)	0.56 (3.8E-07)	0.17 (0.2)	
SLC40A1	ILMN_2053103	exon 8	400 (184-1021)	0.22 (0.06)	0.11 (0.3)	0.55 (7.33E-07)	0.25 (0.03)	0.92 (1.5E-29)

[a] Gene expression is presented in relative fluorescence units.
[b] P-values are written in parentheses.
